# Regional Strain Score as Prognostic Marker of Cardiovascular Events From the Multi-Ethnic Study of Atherosclerosis (MESA)

**DOI:** 10.3389/fcvm.2022.870942

**Published:** 2022-05-13

**Authors:** Theo Pezel, David A. Bluemke, Colin O. Wu, João A. C. Lima, Bharath Ambale Venkatesh

**Affiliations:** ^1^Division of Cardiology, Johns Hopkins Hospital, School of Medicine, Johns Hopkins University, Baltimore, MD, United States; ^2^Department of Cardiology, Lariboisiere Hospital – APHP, INSERM UMRS 942, University of Paris, Paris, France; ^3^University of Wisconsin School of Medicine and Public Health, Madison, WI, United States; ^4^Division of Intramural Research, National Heart Lung and Blood Institute, Bethesda, MD, United States

**Keywords:** cardiac magnetic resonance, regional strain, heart failure, coronary heart disease, Multi-Ethnic Study of Atherosclerosis (MESA)

## Abstract

**Background:**

Left ventricular (LV) circumferential strain (Ecc) is an accurate indicator of regional myocardial function, particularly using the regional Ecc or layer-specific strain.

**Aim:**

This study aimed to investigate the prognostic value of a regional strain score (RSS) for predicting the incident of heart failure (HF) and coronary heart disease (CHD) in a population without a history of cardiovascular disease at baseline.

**Materials and Methods:**

Data from participants in the Multi-Ethnic Study of Atherosclerosis (MESA) who underwent tagged magnetic resonance imaging for strain determination were analyzed. Using −17% and −10% as Ecc cut-offs, each segment was rated from 0 to 2 points according to the Ecc value of each layer. The endo-Ecc, mid-Ecc, and epi-Ecc values from the 16-segment model were used to calculate three RSS: Endo-, Mid-, and Epi-RSS, respectively, which were defined as a percentage of good LV regional function. The Intramyocardial-RSS was the sum of these three RSS. Cox proportional hazard models were used to evaluate the association between each RSS and incident HF and hard CHD.

**Results:**

Among the 1,506 participants (63.3 ± 9.4 years, 54.6% men), 122 cases of hard CHD and 91 cases of HF were observed [median (IQR) follow-up 15.9 (12.9–16.6) years]. After adjustment, Mid-, Epi-, and Intramyocardial-RSS values <50% were independently associated with HF [adjusted HR 1.43; 95% CI (1.08–2.87), *p* = 0.004; HR 1.80; 95% CI (1.12–3.07), *p* < 0.001; and HR 2.01; 95% CI (1.19–3.20), *p* < 0.001]. After adjustment, Endo-, Mid-, Epi-, and Intramyocardial-RSS <50% were also independently associated with hard CHD [adjusted HR 1.31; 95% CI (1.03–1.51), *p* = 0.04; HR 1.79; 95% CI (1.26–2.57), *p* < 0.001; HR 2.03; 95% CI (1.45–3.40), *p* < 0.001; and HR 2.28; 95% CI (1.51–3.53), *p* < 0.001].

**Conclusions:**

Layer-specific regional Ecc, assessed by RSS, provides a robust, independent predictive value for incident HF and hard CHD in asymptomatic participants without any history of previous clinical cardiovascular disease.

**Clinical Trial Registration:**

Unique identifier: NCT00005487.

## Introduction

Cardiovascular mortality due to coronary heart disease (CHD) and heart failure (HF) has recently increased, and associated healthcare costs are expected to double within the next 15 years in the United States (1). The prevalence of CHD and HF are ~6–7% and 1–2%, respectively, of the adult population in developed countries, rising to ≥30% and ≥10%, respectively, among people >70 years of age (1, 2). Given this significant medico-economic burden, it is imperative to develop accurate tools for stratifying the cardiovascular events risk of participants in primary prevention.

To address this issue, several left ventricular (LV) structural and functional parameters have been assessed by cardiovascular magnetic resonance (CMR) and have shown prognostic value in predicting the occurrence of CHD and HF (3–5). Traditionally, the left ventricular ejection fraction (LVEF) has been used as a global index of LV systolic function (2), and several studies support the hypothesis that asymptomatic reduced LVEF is related to the future development of HF and coronary events (6–8). However, LVEF is not a direct measure of myocardial contractility (9), because it is affected by LV geometry and loading conditions and may remain unchanged in affected participants until the underlying disease process is advanced. To address this limitation, global and regional circumferential strain (Ecc) was proposed as a sensitive index of LV myocardial function and described as an earlier marker of incipient myocardial dysfunction (9, 10).

Cardiac-tagged magnetic resonance imaging (MRI) is considered the gold standard of non-invasive myocardial imaging used to measure myocardial strain (10, 11). While more advanced methods of strain calculation have been developed, harmonic phase (HARP) provides an easy and fast method of accurate strain measurement that can be applied clinically and in the subclinical research environment (12, 13). The assessment of global Ecc using tagging has been shown to be an independent prognosticator of cardiovascular events (14, 15). More recently, with an unselected cohort of 539 consecutive patients, Mordi et al. showed that the global Ecc measured by CMR tagging had incremental independent prognostic value for the prediction of cardiovascular events that was greater than that of traditional risk factors, LVEF, and late gadolinium enhancement (16). However, few studies have assessed the long-term prognostic value of the LV regional Ecc assessed by CMR tagging compared to LVEF, traditional risk factors, and global Ecc in asymptomatic participants without a history of cardiovascular disease (CVD) (17, 18).

The myocardial structure is heterogeneous, with layer-specific fiber orientations ranging from largely circumferential at the mid-wall to more oblique at the endocardium and epicardium (19). Beyond the comparison of LV global and regional strains, some studies have recently emphasized the concept of “layer-specific strain,” defined by the fact that global strains measured in the endocardium, epicardium, or mid-wall using CMR are not equivalent for the identification of systolic dysfunction or cardiovascular outcomes (17).

Therefore, we theorized that the regional circumferential myocardial strain could be a more accurate indicator for stratifying the risk of incident HF and CHD among healthy participants, and that specific analysis of the three myocardial layers could further improve its prognostic value. Based on this rationale, we designed an analysis to assess the long-term prognostic value of the layer-specific regional circumferential myocardial strain, using a regional strain score (RSS), measured by CMR tagging in predicting the occurrence of incident HF and CHD in the Multi-Ethnic Study of Atherosclerosis (MESA).

## Materials and Methods

### Study Population

The MESA is a prospective, population-based, multi-ethnic (White, African American, Chinese, and Hispanic) cohort study of subclinical CVD. The study design details have been previously described (20). In summary, between 2000 and 2002, 6,814 men and women aged 45–84 years who were free of clinical CVD at enrollment were recruited from six US field centers (Baltimore, MD; Chicago, IL; Forsyth County, NC; Los Angeles County, CA; Northern Manhattan, NY; and St Paul, MN). As part of the baseline examination, 5,004 (73%) participants underwent comprehensive CMR studies. Of those, 1,773 participants were randomly selected to undergo tagged CMR for myocardial Ecc measurement as an ancillary study protocol at the time of the conventional CMR (*n* = 1,481) or during a separate examination (*n* = 292). The clinical characteristics of this subcohort were similar to those of the entire MESA cohort, except for having a lower body mass index. Among the subcohort, four cases were excluded due to cardiac events reported to have happened before the tagged CMR. The methodology for collecting baseline characteristics is detailed in Supplementary File 1. All participants provided written informed consent. All study protocols were approved by the institutional review boards of each participating field center. The study was conducted in accordance with the Declaration of Helsinki.

**Figure 1:**
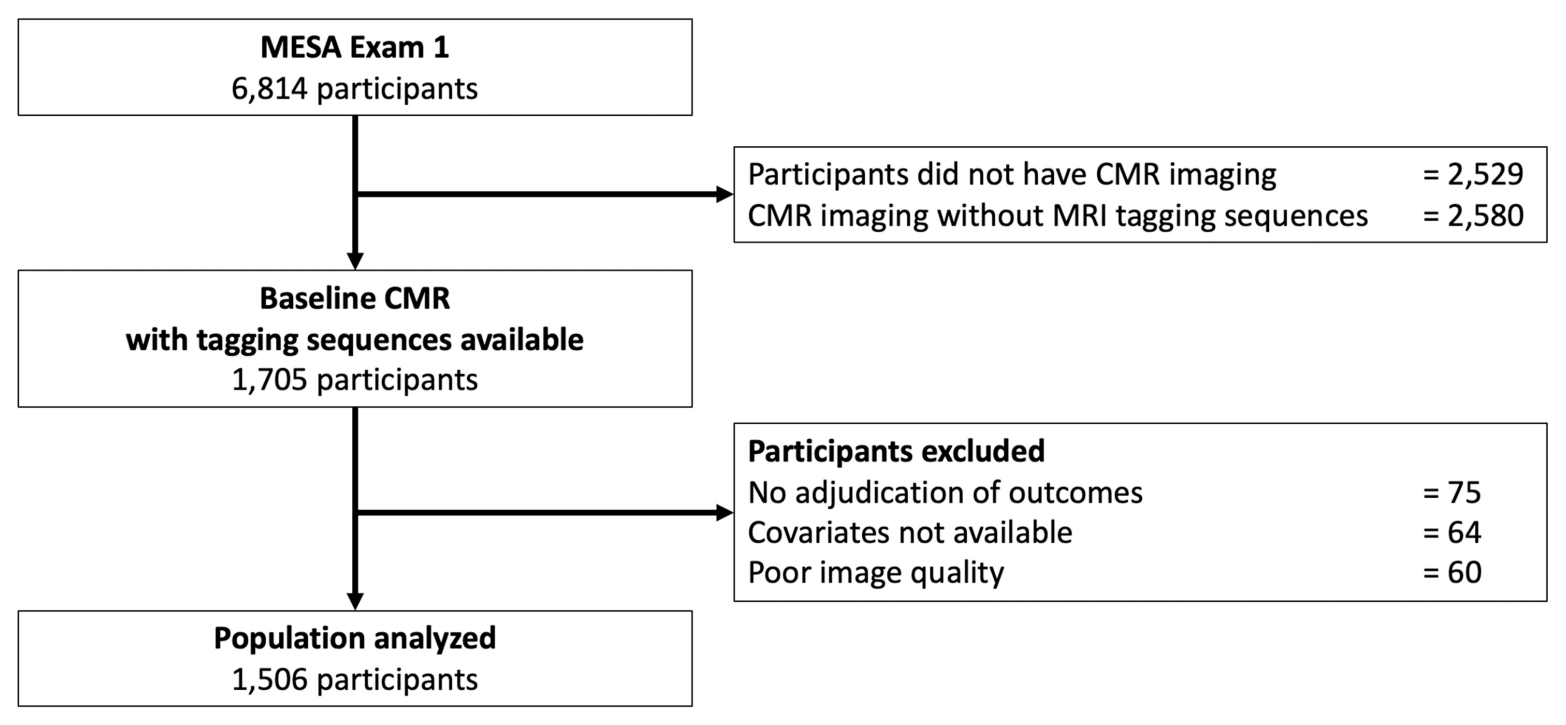
Flowchart of the study. CMR, cardiovascular magnetic resonance; MRI, magnetic resonance imaging.

A flowchart of the MESA population investigated in the current study is depicted in Figure 1.

Of the 4,285 participants who underwent CMR examination, 1,705 participants agreed to a slightly longer CMR examination to accommodate MRI tagging sequences. Of these 1,705 participants, 75 participants had no follow-up for cardiovascular events, 60 had missing images or insufficient image quality with ≥1 segment without well-defined peak Ecc due to significant noise, and 64 had missing covariates. This resulted in a final cohort of 1,506 participants available for the analysis of the proposed study.

### CMR Protocol

Images were acquired using whole-body scanners (1.5CVi, General Electric Medical Systems, Waukesha, WI; Sonata/Symphony Siemens Medical Solutions, Germany) and an electrocardiogram (ECG)-triggered segmented k-space fast spoiled gradient-echo (SPGR or FLASH) pulse sequence during breath-holds. After completing cine CMR using fast gradient-echo imaging for assessment of LV mass and geometry (21), three tagged short axis slices (base to apex) were obtained with an image plane distance of 5–8 mm apart. The slices were positioned to be roughly in the middle of the base, mid-ventricle, and apex, based on four- and two-chamber cine images. Parallel striped tags were prescribed in two orthogonal orientations (0° and 90°) using an ECG-triggered fast gradient-echo sequence with spatial modulation of magnetization (SPAMM), and after they were superimposed as grid images. The tagged CMR image parameters were as follows: field of view 40 cm; slice thickness 8–10 mm; repetition time 3.5–7.2 ms; echo time 2.0–4.2 ms; flip angle 10–12°; matrix size 256 × 96 to 140; temporal resolution 20–40 msec; and tag spacing 7 mm. The detailed protocol used for tagged MRI studies has been previously described (11, 22).

### CMR Image Analysis

All images were read at the central MESA cardiac MRI review center at Johns Hopkins University. LV mass, volumes, and ejection fraction (EF) were determined for each participant using dedicated commercially available software (MASS, 4.2 Medis, the Netherlands) (Supplementary File 2). Short-axis tagged slices were analyzed using the harmonic phase method (HARP commercial v. 3.0, Myocardial Solutions/Diagnosoft, Morrisville, NC) to assess strain (23). A custom user interface built in MATLAB (MathWorks, Natick, MA) was then used to identify segmental and global strain and strain rate peaks. Peak regional systolic Ecc was determined in 16 segments from three LV short-axis slices at the basal, mid-ventricular, and apical levels according to the model of the American Heart Association (24). For each segment, Ecc was determined in the sub-endocardial, mid-wall, and sub-epicardial layers (11, 17). The intraclass correlation coefficients for inter-observer and intra-observer agreement for peak systolic mid-wall Ecc were 0.80 and 0.84, respectively, in studies with good tag persistence, and 0.74 and 0.82, respectively, in those with fair tag persistence (22).

By convention, systolic Ecc, which denotes circumferential shortening, is normally negative; less negative Ecc values reflect decreased regional function. Global peak strain was calculated as the average of the peak strain observed in each segment. Therefore, global Ecc, endo-Ecc, mid-Ecc, and epi-Ecc were defined as the averages of the 16 segments, endo-LV segments, mid-LV segments, and epi-LV segments, respectively. In concordance with prior studies, we considered an Ecc value in any segment that was <-17% as normal (25, 26), and an Ecc value in any segment that was more than −10% as severe dysfunction (25).

### Regional Strain Score

Using the two Ecc cut-offs of −17and −10%, which have been published (25, 26), each segment was rated from 0 to 2 points according to the Ecc value of each layer to grade the LV regional myocardial function for the 16-segment model as follows: (i) 0 points if Ecc was more than −10% for severe regional dysfunction; (ii) 1 point if Ecc was between −17 and −10% for moderate regional dysfunction; and (iii) 2 points if Ecc was <-17% for good regional function. Then, using endo-Ecc, mid-Ecc, and epi-Ecc for the 16-segment model, we defined Endo-, Mid-, and Epi-RSS, respectively, as three indexes of segmental myocardial function ranging from 0 to 32 points. To summarize the overall regional myocardial function, we defined Intramyocardial-RSS as a score ranging from 0 to 96 points, which corresponded to the sum of the Endo-, Mid-, and Epi-RSS. It is worth noting that the Endo-, Mid-, Epi-, and Intramyocardial-RSS values were expressed as percentages, and a higher Endo-, Mid-, Epi-, or Intramyocardial-RSS value indicated better LV regional function.

### Outcomes

The MESA study outcome ascertainment protocols have been described in detail and are available online (www.mesa-nhlbi.org). Cardiovascular endpoints of interest were incident HF and hard CHD. In addition to MESA follow-up examinations every 2 years, a telephone interviewer contacted each participant (or their representative) every 9–12 months to inquire about interim hospital admissions, cardiovascular outpatient diagnoses, and deaths. Two physicians reviewed all records for independent endpoint classification and the assignment of event dates. Criteria for hard CHD outcomes included myocardial infarction, resuscitated cardiac arrest, and death from coronary disease. CHD death included myocardial infarction, chest pain within the 72 h before death, or a history of CHD and the absence of a non-cardiac cause of death. Criteria for HF as an endpoint included symptomatic HF diagnosed by a physician for a patient receiving medical treatment for HF and (1) pulmonary edema/congestion by chest X-ray and/or (2) dilated ventricle or poor LV function by echocardiography or ventriculography, or evidence of LV diastolic dysfunction. A detailed description of the criteria used for each endpoint is provided in Supplementary File 3. If the first cardiovascular event claim occurred before the baseline study, the participant was excluded from the analysis.

### Statistical Analyses

Baseline characteristics are presented as mean ± standard deviation (SD) or median [interquartile range (IQR)] for continuous variables and as counts and percentages for categorical variables. Comparisons employed the χ^2^ or Fisher's exact test for categorical variables and the Student's *t*-test or Mann–Whitney–Wilcoxon test, as appropriate, for continuous variables. We used Endo-, Mid-, Epi-, and Intramyocardial-RSS as independent variables. The survival tree method was used to determine the cut-off to transform continuous Endo-, Mid-, Epi-, and Intramyocardial-RSS into binary variables with the best predictive value for incident HF and hard CHD. Regarding the global Ecc cut-off, we used −16% for HF and −12% for hard CHD, as previously published (14, 16). We used Cox regression models to study the associations between each RSS and outcomes. The assumption of the proportionality of hazards was confirmed for each model. The overall event-free survival rates were calculated using Kaplan-Meier analysis, and the event rates were compared using the Tarone-Ware test. Two models were proposed to assess the associations between each RSS and outcomes. In Model 1, we adjusted for the following traditional cardiovascular risk factors described in the literature (14, 15): age, gender, ethnicity, heart rate, body mass index, hypertension, diabetes, smoking status, dyslipidemia, and one by one the variables of interest among: global circumferential strain, Endo-RSS, Mid-RSS, Epi-RSS, Intramyocardial-RSS or LVEF. In Model 2, we adjusted for cardiovascular medications, including diuretics, beta-blockers, antiarrhythmic agents, calcium channel blockers (CCB), angiotensin-converting enzyme (ACE) inhibitors, angiotensin receptor blocker (ARB), antiplatelet or anticoagulation agents, and one by one the variables of interest among: global circumferential strain, Endo-RSS, Mid-RSS, Epi-RSS, Intramyocardial-RSS or LVEF. Of note, hard CHD was added as a time-dependent covariate in all models for predicting incident HF. Model discrimination was assessed with Harrell's C-statistic. A two-tailed *p*-value <0.05 was considered statistically significant. All data were analyzed using *R* software, version 3.6.1 (R Project for Statistical Computing).

## Results

### Study Population

Among the 1,705 MESA participants with baseline CMR studies that included MRI tagging sequences, 1,506 (88.3%) had tagging, covariates, and outcome data available (mean age 63.3 ± 9.4 years and 54.6% men). Among these 1,506 participants, 39.7% had hypertension, 12.2% had diabetes mellitus, 11.2% were current smokers, and the mean body mass index was 27.6 ± 4.7 kg/m^2^. The population characteristics of all the eligible MESA participants at baseline are described in Supplementary File 4. Of note, there was a lower rate of men and a higher rate of hypertension among the eligible MESA participants at baseline (*n* = 6,814) than in the participants of the current study (*n* = 1,506). The baseline characteristics of the study population, divided into those who developed incident HF (*n* = 91, 6.0%) and hard CHD (*n* = 122, 8.1%) over a median (1st quartile−3rd quartile) follow-up period of 15.9 (12.9–16.6) years, are presented in Table 1. When all pre-specified clinical events were combined, 207 (13.7%) participants had experienced a cardiovascular event. Participants with incident cardiovascular events were older, likelier to be men, and had a higher frequency of diabetes mellitus and hypertension with higher systolic and diastolic blood pressure levels than participants without cardiovascular events. LV functional parameters were globally lower and the LV mass-to-volume ratio was higher in participants who experienced cardiovascular events than in those without cardiovascular events.

**Table 1:** Population characteristics of participants at baseline before occurrence of events by incident event categories.

**Baseline characteristics**	**All patients** ** (*n* = 1,506)**	**No Event** ** (*n* = 1,299)**	**Incident HF** ** (*n* = 91)**	**Hard CHD** ** (*n* = 122)**
Age, years	63.3 ± 9.4	62.7 ± 9.7	**68.2** **±9.2**	**66.4** **±9.9**
Men, n (%)	822 (54.6)	694 (53.4)	**53 (58.7)**	**75 (61.5)**
Ethnicity (Ca/Ch/AA/Hi), %	31/14/27/28	39/13/26/22	**45/11/31/19**	40/10/24/26
Hypertension, *n* (%)	598 (39.7)	460 (35.4)	**64 (70.7)**	**74 (60.8)**
Systolic blood pressure, mmHg	128 ± 21	127 ± 21	**139** **±23**	**135** **±21**
Diastolic blood pressure, mmHg	72 ± 10	72 ± 10	**74** **±13**	**75** **±12**
Body mass index, kg/m^2^	27.6 ± 4.7	27.5 ± 5.0	**29.3** **±5.3**	**28.3** **±4.9**
Diabetes mellitus, *n* (%)	183 (12.2)	132 (10.2)	**23 (25.8)**	**28 (23.1)**
Smoking status, *n* (%)	167 (11.2)	136 (10.5)	11 (12.6)	**20 (16.4)**
Heart rate, bpm	62 ± 9	62 ± 9	**65** **±10**	**64** **±10**
Total cholesterol, mg/dl	194 ± 35	194 ± 36	**190** **±32**	194 ± 36
HDL cholesterol, mg/dl	50 ± 14	50 ± 15	**49** **±14**	**47** **±15**
GFR^*^, ml/min/1.73m^2^	80.8 ± 18.2	81.8 ± 15.6	**72.9** **±20.3**	**76.1** **±19.1**
Chronic kidney disease^†^, n (%)	140 (9.2)	91 (7.0)	**25 (27.2)**	**24 (19.7)**
NT-proBNP, pg/ml	59 (26–118)	40 (13–69)	**164 (87–354)**	**98 (63–303)**
LV function or geometry
LV EDVi, ml/m^2^	70.0 ± 13.1	68.9 ± 13.3	**73.7** **±19.4**	68.3 ± 16.5
LVEF, %	62.4 ± 6.9	62.6 ± 5.9	**58.3** **±9.2**	**62.3** **±7.2**
LV stroke, ml/m^2^	43.9 ± 10.1	44.1 ± 10.2	**40.2** **±11.9**	44.0 ± 11.5
LV mass index, g/m^2^	66.6 ± 12.3	65.9 ± 12.4	**73.6** **±17.9**	**68.8 ± 16.3**
LV MVR, g/ml	0.98 ± 0.18	0.97 ± 0.17	**1.03** **±0.22**	**1.02** **±0.20**
Cardiovascular medication at baseline, *n* (%)
Any ACE inhibitors/ARBs	254 (16.9)	189 (14.5)	**21 (23.1)**	**44 (36.1)**
Any beta-blockers	142 (9.5)	104 (8.0)	**18 (19.8)**	**20 (16.4)**
Any calcium channel blockers	220 (14.6)	176 (13.5)	**19 (20.9)**	**25 (20.5)**
Any diuretics	198 (13.1)	142 (10.9)	**27 (29.7)**	**29 (23.8)**
Aspirin	412 (27.4)	342 (26.3)	**27 (29.7)**	**43 (35.2)**
Any anticoagulation agents	8 (0.5)	6 (0.4)	1 (1.1)	1 (0.8)
Any lipid-lowering medication	266 (17.7)	224 (17.2)	**17 (18.5)**	**25 (20.3)**

*The comparisons with the no event population that were statistically significant with p < 0.05 are shown in bold type*.

* *Glomerular filtration rate (GFR) was calculated by chronic kidney disease epidemiology collaboration (CKD-EPI) method*.

*^†^Chronic kidney disease was defined by Glomerular filtration rate <60 ml/min/1.73 m^2^*.

*AA, African American; ACE, angiotensin-converting enzyme; ARB, angiotensin II receptor blocker; Ca, Caucasian; CHD, coronary heart disease; Ch, Chinese American; HDL, high-density lipoprotein; HF, heart failure; Hi, Hispanic; EDVi, end-diastolic volume indexed; ESVi, end-systolic volume indexed; LV, left ventricle; LVEF, left ventricle ejection fraction; MVR, mass-to-volume ratio; NT-proBNP, N-terminal prohormone of brain natriuretic peptide*.

### Regional Strain Scores

**Figure 2:**
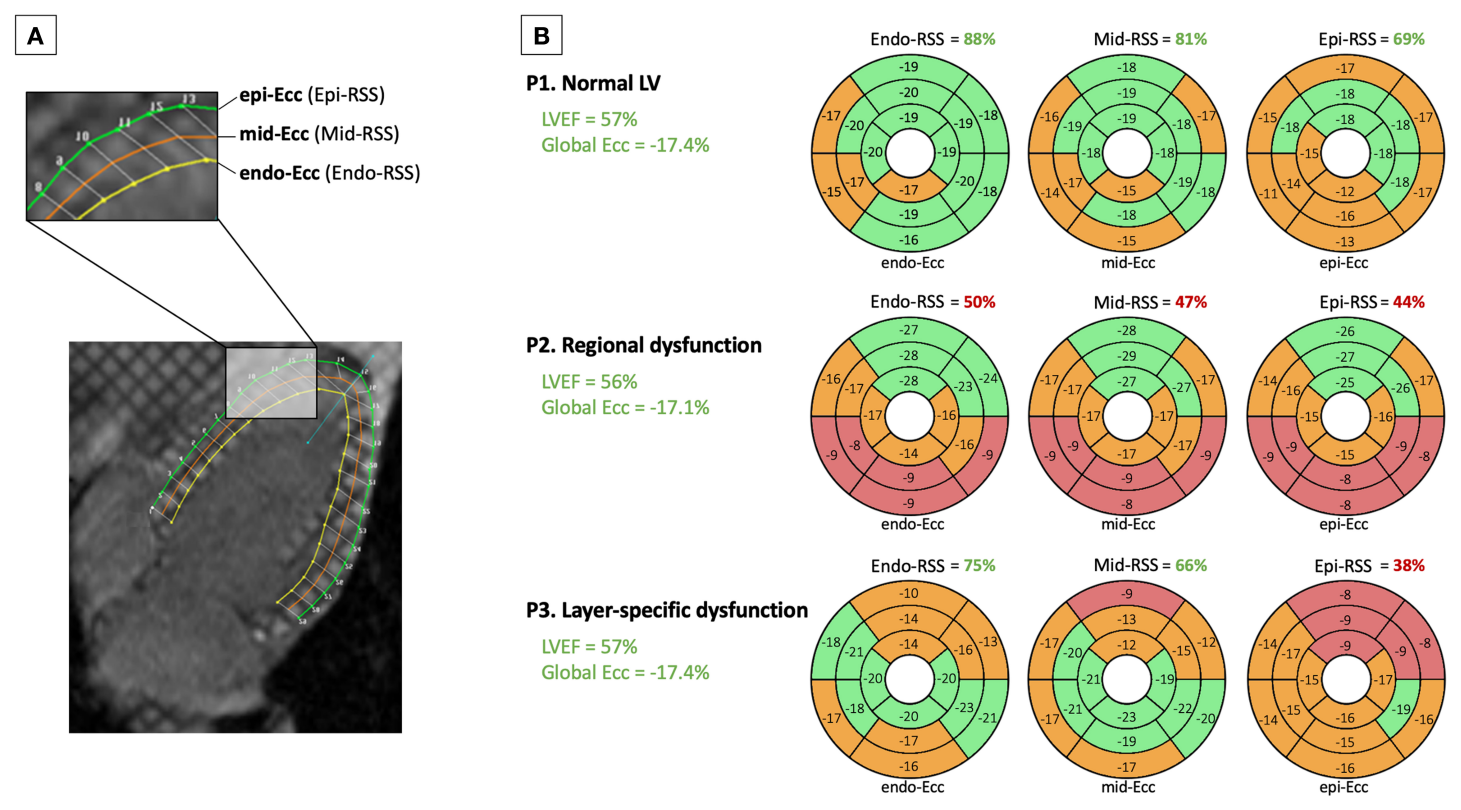
Schematic comparison of variations in the RSS used in different subclinical pathophysiological settings. **(A)** Illustrates the method used to assess global, regional, and layer-specific Ecc using short-axis tagged magnetic resonance images. Grid lines (tags) are visible, and contours drawn at three myocardial levels [green [epicardial], orange [mid-myocardial], and yellow [endocardial]] allow tracking of myocardial motion and Ecc. Endo-RSS, Mid-RSS, and Epi-RSS were defined using endo-Ecc, mid-Ecc, and epi-Ecc for the 16-segment model, respectively, as three indexes expressed as a percentage of good LV regional function. **(B)** Shows three patients from this cohort who had the same normal LVEF value (57%) and global Ecc (−17%). Patient 1 (P1) had a normal regional Ecc, assessed using Mid-RSS as a score of the mid-Ecc (81%). Patient 2 (P2) had a normal global Ecc (−17.1%); however, he had a regional dysfunction defined by an altered regional Ecc, with all three RSS <50%. Patient 3 (P3) had normal Endo-RSS (75%) and Mid-RSS (66%); however, he had a layer-specific dysfunction defined by a reduced Epi-RSS <50%. These three patients had different RSS values and significantly different risk levels of cardiovascular events that were not detected when using the LVEF or global Ecc value alone. A higher RSS value reflects better regional LV function, expressed as a percentage of good myocardial function.

For the entire study population, the mean Endo-, Mid-, Epi-, and Intramyocardial-RSS were 70.9 ± 15.2%, 67.1 ± 16.1%, 57.4 ± 16.8%, and 65.1 ± 15.2%, respectively (Supplementary File 5). There were no significant differences between women and men (Supplementary File 6). The theoretical framework underlying RSS used in various subclinical pathophysiological settings is illustrated in the Figure 2.

### Regional Strain Scores and Incident HF

**Figure 3:**
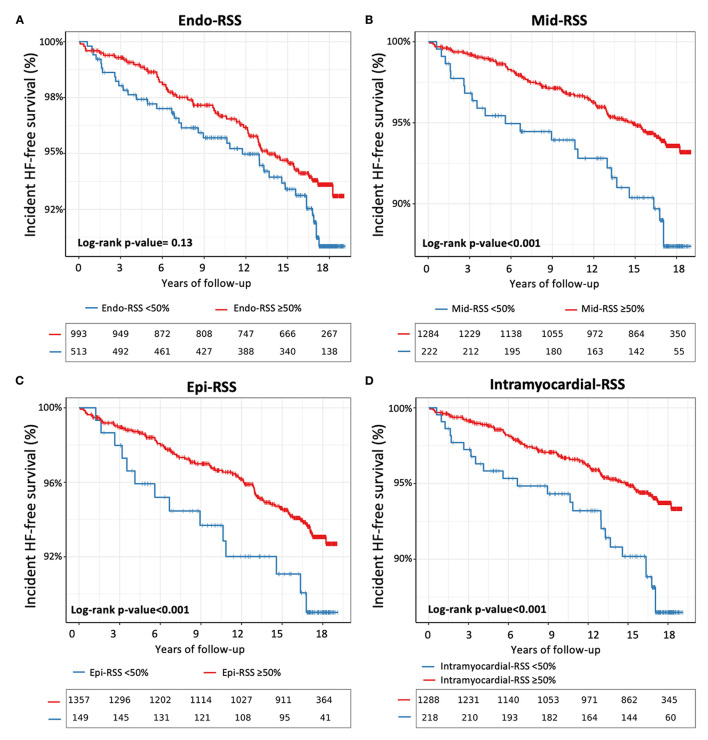
Kaplan–Meier survival curves for incident HF stratified by Endo-RSS <50% **(A)**, Mid-RSS <50% **(B)**, Epi-RSS <50% **(C)**, and intramyocardial-RSS <50% **(D)**.

The results from the unadjusted and adjusted Cox proportional hazard models for the Endo-, Mid-, Epi-, and Intramyocardial-RSS are presented in Table 2. Using an optimal cut-off point to predict incident HF defined by the survival tree method (Supplementary File 7), Mid-, Epi-, and Intramyocardial-RSS <50% were associated with incident HF [hazard ratio, HR 1.69; 95% confidence interval, CI (1.14–3.17), HR 2.01; 95% CI (1.25–3.22), and HR 2.12; 95% CI (1.38–3.55), respectively; all *p* < 0.001] (Figure 3). Endo-RSS was not associated with incident HF (*p* =0.13). After adjustment for all traditional risk factors (Model 1), Mid-, Epi-, and Intramyocardial-RSS <50% remained independently associated with incident HF [adjusted HR 1.43; 95% CI (1.08–2.87), *p* = 0.004; HR 1.80; 95% CI (1.12–3.07), *p* < 0.001; and HR 2.01; 95% CI (1.19–3.20), *p* < 0.001]. The multivariable models including Mid-, Epi-, or Intramyocardial-RSS additionally to traditional risk factors showed significant improvement in model discrimination compared to the multivariable model with only traditional risk factors for predicting incident HF (C-statistic: 0.76 vs. 0.74; C-statistic: 6 0.78 vs. 0.74; and C-statistic: 0.79 vs. 0.74, respectively). Intramyocardial-RSS also demonstrated better discrimination for incident HF than the multivariable model with global Ecc or LVEF (C-statistic: 0.79 vs. 0.74 and 0.76, respectively, Table 2). After adjustment for all cardiovascular medications (Model 2), Mid-, Epi-, and Intramyocardial-RSS <50% were associated with incident HF [HR 1.71; 95% CI (1.13–3.19), HR 1.97; 95% CI (1.20–3.20), and HR 2.03; 95% CI (1.33–3.49), respectively; all *p* < 0.001; Supplementary File 7].

**Table 2:** Univariable and multivariable analysis for incident HF (*N* = 1,506).

	**Univariable analysis**		**Multivariable analysis**			
	**Hazard ratio (95% CI)**	***p*-value**	**Hazard ratio (95% CI)**	***p*-value**		**C-statistics (95% CI)**
Age	1.06 (1.03–1.08)	**<0.001**	1.01 (0.98–1.05)	0.44	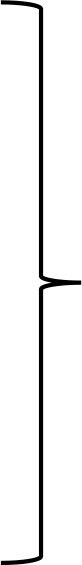	
Men	1.57 (1.02–2.42)	**0.039**	2.00 (1.30–3.18)	**<0.001**		
Ethnicity	1.23 (0.79–1.92)	0.35	1.15 (0.68–1.43)	0.71		
Body mass index	1.05 (1.01–1.00)	**0.008**	1.01 (0.98–1.06)	1.000		0.74 (0.70–0.77)
Hypertension	2.81 (1.79–4.41)	**<0.001**	1.34 (0.78–2.27)	0.65		
Diabetes mellitus	1.99 (1.20–3.31)	**<0.001**	1.79 (1.18–2.72)	**0.004**		
Current smoking	1.25 (0.62–1.79)	0.83	1.21 (0.60–1.72)	0.88		
Hypercholesterolemia	1.10 (0.77–1.58)	0.55	0.90 (0.60–1.34)	0.71		
Incident hard CHD	1.24 (0.70–2.87)	0.72	1.12 (0.61–1.76)	0.82		
Global circumferential strain (continuous)	1.03 (1.01–1.04)	**<0.001**	1.01 (0.98–1.04)	0.77		0.74 (0.70–0.77)
Global circumferential strain >-16%	1.58 (1.07–2.49)	**0.002**	1.18 (1.03–1.59)	**0.016**		0.74 (0.70–0.77)
Endo–RSS <50%	1.38 (0.91–2.09)	0.13	1.30 (0.92–2.02)	0.56		0.74 (0.70–0.77)
Mid-RSS <50%	1.69 (1.14–3.17)	**<0.001**	1.43 (1.08–2.87)	**0.004**		0.76 (0.72–0.79)
Epi-RSS <50%	2.01 (1.25–3.22)	**<0.001**	1.80 (1.12–3.07)	**<0.001**		0.78 (0.74–0.80)
Intramyocardial-RSS <50%	2.12 (1.38–3.55)	**<0.001**	2.01 (1.19–3.20)	**<0.001**		0.79 (0.75–0.81)
LVEF, %	0.70 (0.63–0.78)	**<0.001**	0.72 (0.66–0.80)	**<0.001**		0.76 (0.72–0.79)
LVEF >50%	0.61 (0.40–0.97)	**0.042**	0.64 (0.43–1.07)	0.06		0.76 (0.72–0.79)

*Adjusted model 1 included: age, gender, ethnicity, body mass index, hypertension, diabetes mellitus, current smoking, hypercholesterolemia, incident hard CHD as time dependent covariate, and one by one the variables of interest among: global circumferential strain, Endo-RSS, Mid-RSS, Epi-RSS, Intramyocardial-RSS or LVEF*.

*AA, African American; ACE, angiotensin-converting enzyme; ARB, angiotensin II receptor blocker; Ca, Caucasian; CHD, coronary heart disease; Ch, Chinese American; HDL, high-density lipoprotein; HF, heart failure; Hi, Hispanic; EDVi, end-diastolic volume indexed; ESVi, end-systolic volume indexed; LV, left ventricle; LVEF, left ventricle ejection fraction; MVR, mass-to-volume ratio; NT-proBNP, N-terminal prohormone of brain natriuretic peptide; RSS, regional strain score*.

### Regional Strain Scores and Incident Hard CHD

**Figure 4:**
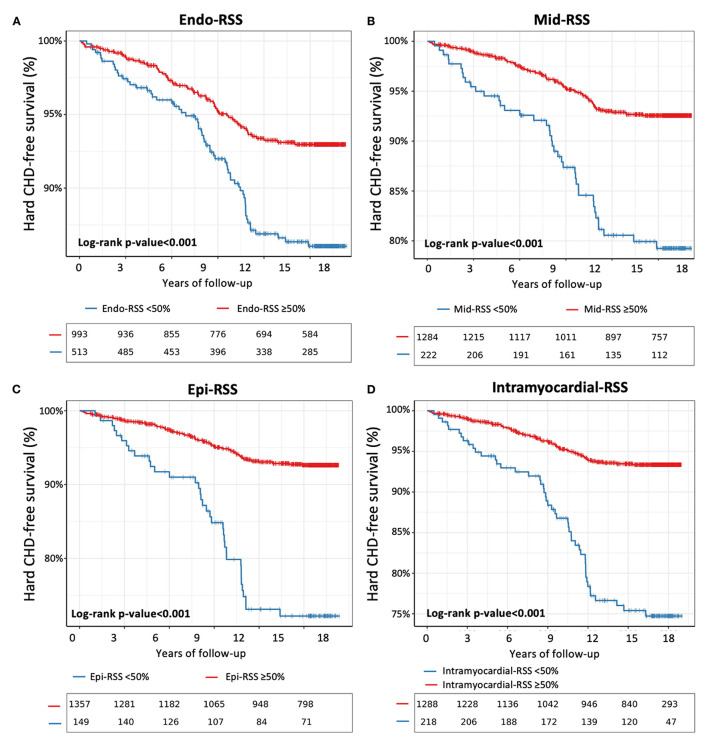
Kaplan–Meier survival curves for incident hard CHD stratified by Endo-RSS <50% **(A)**, Mid-RSS <50% **(B)**, Epi-RSS <50% **(C)**, and intramyocardial-RSS <50% **(D)**.

The results from the unadjusted and adjusted Cox proportional hazard models for the Endo-, Mid-, Epi-, and Intramyocardial-RSS are presented in Table 3. Using an optimal cut-off point to predict hard CHD defined by the survival tree method (Supplementary File 8), Endo-, Mid-, Epi-, and Intramyocardial-RSS <50% were associated with hard CHD [HR 1.33; 95% CI (1.22–1.59), HR 1.85; 95% CI (1.32–2.31), HR 2.32; 95% CI (1.56–3.60), and HR 2.40; 95% CI (1.60–3.74), respectively; all *p* < 0.001] (Figure 4). After adjustment for all traditional risk factors (Model 1), Endo-, Mid-, Epi-, and Intramyocardial-RSS <50% remained independently associated with hard CHD [adjusted HR 1.31; 95% CI (1.03–1.51), *p* = 0.04; HR 1.79; 95% CI (1.26–2.57), *p* < 0.001; HR 2.03; 95% CI (1.45–3.40), *p* < 0.001; and HR 2.28; 95% CI (1.51–3.53), *p* < 0.001]. The multivariable model with Epi- and Intramyocardial-RSS showed significant improvement in model discrimination compared to the multivariable model with traditional risk factors for predicting hard CHD (C-statistic: 0.74 vs. 0.73; and C-statistic: 0.75 vs. 0.73, respectively). Intramyocardial-RSS also demonstrated better discrimination for hard CHD than the multivariable model with global Ecc or LVEF (C-statistic: 0.75 vs. 0.73, Table 3). After adjustment for all cardiovascular medications (Model 2), Endo-, Mid-, Epi-, and Intramyocardial-RSS <50% remained independently associated with hard CHD [adjusted HR 1.45; 95% CI (1.11–1.67); HR 1.83; 95% CI (1.29–2.61); HR 2.06; 95% CI (1.47–3.49); and HR 2.24; 95% CI (1.47–3.46), all *p* <0.001; Supplementary File 9].

**Table 3:** Univariable and multivariable analysis for hard CHD (*N* = 1,506).

	**Univariable analysis**		**Multivariable analysis**			
	**Hazard ratio (95% CI)**	***p*-value**	**Hazard ratio (95% CI)**	***p*-value**		**C-statistics (95% CI)**
Age	1.03 (1.02–1.05)	**<0.001**	1.01 (0.98–1.03)	0.53	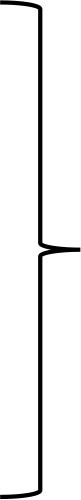	
Men	1.95 (1.44–2.64)	**<0.001**	2.10 (1.36–3.24)	**<0.001**		
Ethnicity	0.82 (0.59–1.15)	0.25	0.81 (0.55–1.18)	0.27		
Body mass index	1.01 (0.99–1.02)	0.48	1.00 (0.97–1.04)	1.00		0.73 (0.69–0.77)
Hypertension	2.08 (1.55–2.78)	**<0.001**	1.35 (0.79–2.29)	0.27		
Diabetes mellitus	2.32 (1.65–3.28)	**<0.001**	1.80 (1.20–2.70)	**0.005**		
Current smoking	0.73 (0.48–1.12)	0.15	0.74 (0.45–1.15)	0.22		
Hypercholesterolemia	1.09 (0.76–1.57)	0.62	0.92 (0.62–1.35)	0.66		
Global circumferential strain (continuous)	1.02 (1.01–1.03)	**0.036**	1.00 (0.99–1.02)	0.67		0.73 (0.69–0.77)
Global circumferential strain >-12%	1.29 (0.73–2.82)	0.61	1.20 (0.67–2.61)	0.72		0.73 (0.69–0.77)
Endo-RSS <50%	1.33 (1.22–1.59)	**<0.001**	1.31 (1.03–1.51)	**0.04**		0.73 (0.69–0.77)
Mid-RSS <50%	1.85 (1.32–2.31)	**<0.001**	1.79 (1.26–2.57)	**<0.001**		0.73 (0.69–0.77)
Epi-RSS <50%	2.32 (1.56–3.60)	**<0.001**	2.03 (1.45–3.40)	**<0.001**		0.74 (0.70–0.78)
Intramyocardial-RSS <50%	2.40 (1.60–3.74)	**<0.001**	2.28 (1.51–3.53)	**<0.001**		0.75 (0.71–0.79)
LVEF, %	0.84 (0.77–0.92)	**0.001**	0.88 (0.82–0.97)	**0.02**		0.73 (0.69–0.77)
LVEF >50%	0.73 (0.50–1.04)	0.07	0.78 (0.52–1.10)	0.23		0.73 (0.69–0.77)

*Adjusted model 1 included: age, gender, ethnicity, body mass index, hypertension, diabetes mellitus, current smoking, hypercholesterolemia, and one by one the variables of interest among: global circumferential strain, Endo-RSS, Mid-RSS, Epi-RSS, Intramyocardial-RSS or LVEF*.

*AA, African American; ACE, angiotensin-converting enzyme; ARB, angiotensin II receptor blocker; Ca, Caucasian; CHD, coronary heart disease; Ch, Chinese American; HDL, high-density lipoprotein; HF, heart failure; Hi, Hispanic; EDVi, end-diastolic volume indexed; ESVi, end-systolic volume indexed; LV, left ventricle; LVEF, left ventricle ejection fraction; MVR, mass-to-volume ratio; NT-proBNP, N-terminal prohormone of brain natriuretic peptide; RSS, regional strain score*.

## Discussion

In this multi-ethnic population of participants, aged from 45 to 84 years, and free of clinical CVD at enrollment, the main findings are: (i) Mid-, Epi-, and Intramyocardial-RSS were independently associated with incident HF after adjusting for traditional risk factors or cardiovascular medications; (ii) Endo-, Mid-, Epi-, and Intramyocardial-RSS were independently associated with incident hard CHD after adjusting for traditional risk factors or cardiovascular medications; (iii) Epi- and Intramyocardial-RSS showed significant improvement in model discrimination compared to the multivariable model with traditional risk factors for predicting both incident HF and hard CHD, and better discrimination than the multivariable model with global Ecc or LVEF. To our knowledge, the prognostic value of such a strain index that combines regional and layer-specific strain, and shows improvement compared to traditional risk factors, global Ecc, and LVEF, has not been previously reported.

The current study shows that regional Ecc provides an incremental prognostic value for predicting the occurrence of HF and hard CHD that exceeds the effectiveness of traditional risk factors. This feature of Ecc (used as a marker for identifying participants at risk of HF or CHD) is consistent with the results of several previous studies (18, 27). Among all fibers in the LV myocardium, circumferential fibers predominate, and circumferential shortening is the major determinant of stroke volume (9). Regarding pathophysiology, it has been shown that impaired regional Ecc may represent a response to increased myocardial wall stress and reflect local abnormalities, such as fibrosis or ischemia due to macro- or microvascular disease. Furthermore, impairments in both circumferential and longitudinal strains have been found to be associated with LV remodeling and macrovascular or microvascular disease in patients with CHD (28). In particular, impaired global Ecc could be related to ischemic remodeling because circumferential function helps in maintaining LV function after impaired longitudinal function (28, 29).

This study also shows that regional myocardial Ecc, assessed using the RSS, provides a better prognostic value than LVEF for the occurrence of HF and hard CHD. These findings are consistent with a prior MESA study (14) in which Choi et al. assessed Ecc data from 1,768 asymptomatic patients who underwent CMR tagging. The authors found that global Ecc provided an incremental prognostic value when added to baseline clinical variables and LVEF, including in patients with preserved LVEF. In our study, beyond the LVEF value, the RSS were better prognostic indicators than the global Ecc for predicting HF and hard CHD. Indeed, some studies have already hypothesized that the assessment of regional Ecc may identify areas of reduced contractility caused by diffuse fibrosis or ischemia not picked up by global Ecc (16).

Recently, Xu et al. emphasized that global strains measured in the endocardium, mid-ventricle, or epicardium are not equivalent for the prediction of HF outcomes (17). Therefore, beyond the comparison between LV global and regional strain, we also investigated the prognostic value of layer-specific Ecc using Endo-, Mid-, and Epi-RSS. After adjusting for all covariates, it was found that Endo-, Mid-, and Epi-RSS were all independently associated with incident hard CHD; only Mid- and Epi-RSS were independently associated with incident HF. Similar results were obtained after adjusting for cardiovascular medications, which reinforces the robustness of these findings. Among these three RSS, Epi-RSS showed significant improvement in model discrimination compared to the multivariable model with traditional risk factors for predicting both incident HF and hard CHD, and better discrimination than the multivariable model with global Ecc or LVEF. All these findings are globally in line with those of a recent study that showed that the endocardium-specific strains had the poorest all-around performance, and are also in agreement with the patterns of preserved endocardium-specific strains and reduced epicardium-specific strains in the HF population (17).

However, regarding the relationships between cardiovascular outcomes and layer-specific strains, other studies have suggested that endocardium-specific strains could also be helpful in stratifying the risk of cardiovascular events (30). Therefore, to capture all the information provided by the three myocardial layers, we developed the Intramyocardial-RSS, which is defined by the sum of the Endo-, Mid-, and Epi-RSS for the 16-segment model. Interestingly, Intramyocardial-RSS showed the greatest improvement in model discrimination compared to the model with traditional risk factors for predicting both incident HF and hard CHD—it outperformed the three other RSS. This finding regarding the better prognostic value of Intramyocardial-RSS could be explain by the fact that this method allows both the measurement of regional strain granularity by segment and by layer, while capturing the entire myocardial information. Finally, early detection of subclinical LV impairment using layer-specific regional strain could pave the way for personalized cardiovascular risk stratification and new therapeutic strategies that might slow or change a person's clinical history, impacting their quality of life and mortality. Further studies could be conducted to evaluate the early pharmacologic effects on the RSS.

### Study Limitations

This study has some limitations. First, the general applicability of these findings may be limited by selection and survivor biases. Indeed, participants had no known CVD at baseline; therefore, the older participants who underwent CMR represent a healthier sample than the general older population. Second, incident HF was not differentiated into HF with preserved or reduced LVEF due to the limited power for sub-analysis, given the low number of events. Third, the exclusion of participants with no adjudicated outcome, unavailable tagging-CMR data, or poor-quality images could have introduced bias into the study. Fourth, we included only Ecc in this study due to the lack of longitudinal strain measurements in the MESA imaging acquisition protocol and less reproducibility of the strain rate compared with circumferential shortening by tagged MRI. Fifth, the current study did not allow comparison of strain measurements obtained by CMR with strain data obtained by echocardiography. Knowing that CMR is not a widely accessible test in routine, the use of RSS as a screening tool in the general population should be investigated in echocardiography. In addition, further studies should also evaluate the incremental prognostic value of RSS compared with other biomarkers such as troponin, NT-proBNP (N-terminal pro-B-type natriuretic peptide) or and diastolic dysfunction parameters using echocardiography. Finally, residual confounding cannot be completely eliminated from this cross-sectional study because only the traditional risk factors assessed at baseline were analyzed in the final models without any utilization of time-varying covariates. In addition, the final multivariate models did not include specific biomarkers such as NTproBNP, GFR, or CMR parameters.

## Conclusion

In a large multi-ethnic population free of clinical CVD at baseline, layer-specific regional Ecc, via RSS, provides a significant, independent prognostic value for the occurrence of HF and hard CHD compared to global Ecc and LVEF. Among the indexes, Epi-RSS and Intramyocardial-RSS provide the best incremental risk prediction compared to traditional risk factors for predicting both incident HF and hard CHD. The results of this study support the hypothesis that layer-specific regional Ecc can be used as an additional parameter for the risk stratification of subclinical HF and CHD among asymptomatic participants without a previous history of heart disease.

## Data Availability Statement

The raw data supporting the conclusions of this article will be made available by the authors, without undue reservation.

## Ethics Statement

The studies involving human participants were reviewed and approved by IRB. The patients/participants provided their written informed consent to participate in this study.

## Author Contributions

TP and BA conceived the study design, performed statistical analyses, analyzed data, and drafted the manuscript with critical revision. All authors contributed to the manuscript and revision, participated in the discussion of the concept of the study, and read and approved the final manuscript.

## Funding

This study received funding from Myocardial solution^®^. The funder was not involved in the study design, collection, analysis, interpretation of data, the writing of this article or the decision to submit it for publication. The Multi-Ethnic Study of Atherosclerosis was supported by contracts 75N92020D00001, HHSN268201500003I, N01-HC-95159, 75N92020D00005, N01-HC-95160, 75N92020D00002, N01-HC-95161, 75N92020D00003, N01-HC-95162, 75N92020D00006, N01-HC-95163, 75N92020D00004, N01-HC-95164, R01 HL127659, 75N92020D00007, N01-HC-95165, N01-HC-95166, N01-HC-95167, N01-HC-95168, and N01-HC-95169 from the National Heart, Lung, and Blood Institute, and by grants UL1-TR000040, UL1-TR-001079, and UL1-TR-001420 from the National Center for Advancing Translational Sciences (NCATS).

## Author Disclaimer

The views expressed in this manuscript are those of the authors and do not necessarily represent the views of the National Heart, Lung, and Blood Institute; the National Institutes of Health; or the U.S. Department of Health and Human Services.

## Conflict of Interest

The authors declare that the research was conducted in the absence of any commercial or financial relationships that could be construed as a potential conflict of interest.

## Publisher's Note

All claims expressed in this article are solely those of the authors and do not necessarily represent those of their affiliated organizations, or those of the publisher, the editors and the reviewers. Any product that may be evaluated in this article, or claim that may be made by its manufacturer, is not guaranteed or endorsed by the publisher.

## Abbreviations

CMR: cardiovascular magnetic resonance
CHD: coronary heart disease
Ecc: circumferential strain
HF: heart failure
LV: left ventricle
LVEF: left ventricular ejection fraction
MESA: Multi-Ethnic Study of Atherosclerosis
RSS: Regional Strain Score.
