# Supplementary material for: Regional Strain Score as Prognostic Marker of Cardiovascular Events From the Multi-Ethnic Study of Atherosclerosis (MESA)
Source: Front Cardiovasc Med. 2022 May 13;9:870942. doi: 10.3389/fcvm.2022.870942 (PMC9136083; doi:10.3389/fcvm.2022.870942)
Supplement: Supplementary file 1 [file Data_Sheet_1.docx]

**SUPPLEMENTARY FILES**

**Table of Contents:**

- Supplementary file 1: Methodology of baseline characteristics collection.
- Supplementary file 2: CMR analysis: left ventricular geometry and chamber performance assessment.
- Supplementary file 3: Definition of outcomes.
- Supplementary file 4: Population characteristics of all MESA participants at baseline.
- Supplementary file 5: Distribution of Endo-, Mid-, Epi- and Intramyocardial-RSS in the overall population.
- Supplementary file 6: Distribution of Endo-, Mid-, Epi- and Intramyocardial-RSS values stratified by gender.
- Supplementary file 7: Univariable and Multivariable Analysis for Incident HF for model 2 (N=1,506).
- Supplementary file 8: Determination of the optimal cut-off to transform Endo-, Mid-, Epi- and Intramyocardial-RSS into a binary variable with the best predictive value for each outcome.
- Supplementary file 9: Univariable and Multivariable Analysis for Hard CHD for model 2 (N=1,506).

**SUPPLEMENTARY FILE 1:**

**Methodology of baseline characteristics collection**

- Standardized questionnaires were used at baseline (Exam 1) to collect information about age, gender, race/ethnic background and cigarette smoking. A medication inventory was used to collect information on prescription and nonprescription medications.
- Cigarette smoking was categorized as current, former or never. Body mass index was calculated as weight divided by height squared (kg/m^2^) with weight measured to the nearest 0.5kg and height to the nearest 0.1 cm. Blood pressure was measured 3 times using a Dinamap model Pro 100 automated oscillometric sphygmomanometer (Critikon; Tampa, FL) while the participants were resting in a seated position. The average of the last two measurements were used in the analysis. Fasting glucose was obtained by a thin-film adaptation of the glucose oxidase method (Johnson & Johnson Clinical Diagnostics, Inc, Rochester, NY). Diabetes mellitus was defined as a fasting glucose of ≥126 mg/dL or use of hypoglycemic medication.
- Blood samples were stored at −70°C and were thawed before testing (maximum of 3 freeze-thaw cycles). NT-proBNP was measured using the Elecsys 2010 system (Roche Diagnostics, Indianapolis, IN). All analyses were performed at a core laboratory (Veteran’s Affairs San Diego Healthcare System, La Jolla, CA). Intra- and interassay coefficients of variation at various concentrations of NT-proBNP were 1.3% and 4.8%, respectively (*Karl, J, et al. Development of a novel, N-terminal-proBNP (NT-proBNP) assay with a low detection limit. Scand J Clin Lab Invest Suppl. 1999;230:177–181*). The analytical measurement range for NT-proBNP was 5 to 35 000 pg/mL.

**SUPPLEMENTARY FILE 2:**

**CMR analysis: left ventricular geometry and chamber performance assessment.**

The endocardial and epicardial myocardial borders were contoured using a semi-automated method (MASS 4.2, Medis, Leiden, the Netherlands). The difference between the epicardial and endocardial areas for all slices was multiplied by the slice thickness and section gap, and then multiplied by the specific gravity of the myocardium to determine the ventricular mass. All of these measurements were performed at end diastole. The papillary muscle mass was included in the LV cavity and excluded from the LV mass measurements.^1^ The stroke volume (SV) was calculated as LV end-diastolic volume (LVEDV) minus end-systolic volume (LVESV). The LV mass index was calculated by the LV mass/body surface area.

References:

1. Bluemke DA, Kronmal RA, Lima JAC, Liu K, Olson J, Burke GL, Folsom AR. The relationship of left ventricular mass and geometry to incident cardiovascular events: the MESA (Multi-Ethnic Study of Atherosclerosis) study. *J Am Coll Cardiol* 2008;**52**:2148–2155.

**SUPPLEMENTARY FILE 3:**

**Definition of outcomes.**

The MESA outcome event ascertainment protocols have been described in detail and are available online ([www.mesa-nhlbi.org](http://www.mesa-nhlbi.org)). In addition to MESA follow-up examinations, a telephone interviewer contacted each participant (or representative) every 9–12 months to inquire about interim hospital admissions, CV outpatient diagnoses, and mortality.

**Incident heart failure (HF)**

Medical records were reviewed and diagnoses of HF events were adjudicated by a panel of MESA physicians using standardized criteria. We used both probable and definite HF events for analysis. Probable HF was defined as a physician diagnosis and a receipt of HF medical treatment. Definite HF required an additional criterion; such as evidence of pulmonary congestion on chest radiography, reduced LV function by echocardiography or ventriculography, or evidence of LV diastolic dysfunction. EF measures were recorded from clinical echocardiography for events diagnosed as HF by MESA cardiac reviewers. The last HF events data was followed-up to December 2017.

**Hard coronary heart disease (CHD)**

Criteria for hard CHD outcomes included:

- ***Myocardial infarction***: the diagnosis of myocardial infarction during the follow-up period required either abnormal cardiac biomarkers (two times upper limits of normal) regardless of pain or ECG findings; evolving Q waves regardless of pain or biomarker findings; or a combination of chest pain, and ST-T evolution or new LBBB, and biomarker levels 1-2 times upper limits of normal.
- ***Resuscitated cardiac arrest***: reviewers classified resuscitated cardiac arrest when a patient successfully recovered from a full cardiac arrest through cardiopulmonary resuscitation (including cardioversion), or evidence of ischemia by stress tests or by resting ECG. We considered coronary revascularization or a physician diagnosis of angina or CHD, in the absence of symptoms, to not be angina.
- ***CHD death***: definite fatal CHD required a documented myocardial infarction within the previous 28 days, chest pain within the 72 hours before death, or a history of CHD, and required the absence of a known non-atherosclerotic or non-cardiac cause of death. If the definite fatal CHD criteria were not met, possible fatal CHD could be assigned with an underlying cause of death consistent with fatal CHD and required the absence of a known non-atherosclerotic or non-cardiac cause of death.

**SUPPLEMENTARY FILE 4:**

**Population characteristics of all MESA participants at baseline.**

| **Parameters** | **All participants included**  **(n=1,506)** | | **All participants eligible at baseline**  **(n=6,814)** | |
| --- | --- | --- | --- | --- |
|  |  | |  | |
| Age, years | 63.3 ± 9.4 | | 62.1 ± 10 | |
| Men, n (%) | 822 (54.6) | | **3209 (47.1)** | |
| Ethnicity (Ca/Ch/AA/Hi), % | 31/14/27/28 | | **39/12/28/22** | |
| Hypertension, n (%) | 598 (39.7) | | **3059 (44.9)** | |
| Systolic blood pressure, mmHg | 128 ± 21 | | 126 ± 22 | |
| Diastolic blood pressure, mmHg | 72 ± 10 | | 72 ± 10 | |
| Body mass index, kg/m^2^ | 27.6 ± 4.7 | | 28.3 ± 5.5 | |
| Diabetes mellitus, n (%) | 183 (12.2) | | 859 (12.6) | |
| Smoking status, n (%) | 167 (11.2) | | 886 (13.0) | |
| Heart rate, bpm | 62 ± 9 | | 63 ± 10 | |
| Total cholesterol, mg/dl | 194 ± 35 | | 194 ± 36 | |
| HDL cholesterol, mg/dl | 50 ± 14 | | 51 ± 15 | |
|  |  |  |  |  |

The comparisons that were statistically significant with p<0.05 are shown in bold type.

Abbreviations: AA: African American; Ca: Caucasian; CHD: coronary heart disease; Ch: Chinese American; CVD: cardiovascular disease; HDL: high-density lipoprotein; Hi: Hispanic.

**SUPPLEMENTARY FILE 5:**

**Distribution of Endo-, Mid-, Epi- and Intramyocardial-regional strain score (RSS)** **in the overall population.**

The Endo-, Mid-, Epi- and Intramyocardial-RSS values were expressed as a percentage, and a higher Endo-, Mid-, Epi- or Intramyocardial-RSS expressed a better LV regional function.

*Abbreviations: RSS: regional strain score.*

**
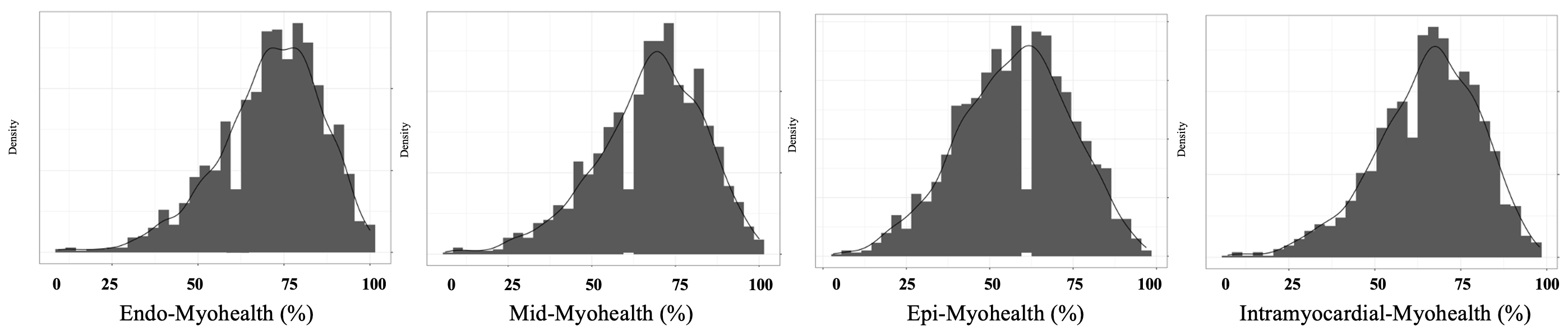
**

**SUPPLEMENTARY FILE 6:**

**Distribution of Endo-, Mid-, Epi- and Intramyocardial-regional strain score (RSS)** **values stratified by gender.**

| RSS | **Women**  **(n=684)** | **Men**  **(n=822)** | **p-value** |
| --- | --- | --- | --- |
| Endo-RSS | 71.4 ± 16.8 | 70.2 ± 15.4 | p=0.08 |
| Mid-RSS | 67.4 ± 17.0 | 66.9 ± 15.8 | p=0.73 |
| Epi-RSS | 57.8 ± 16.7 | 57.1 ± 15.4 | p=0.66 |
| Intramyocardial-RSS | 65.4 ± 16.7 | 64.9 ± 15.4 | p=0.72 |

*Abbreviations: RSS: regional strain score.*

**SUPPLEMENTARY FILE 7:**

**Univariable and Multivariable Analysis for Incident HF for model 2 (N=1,506).**

|  | **Univariable analysis** | |  | **Multivariable analysis** | | |
| --- | --- | --- | --- | --- | --- | --- |
|  | **Hazard Ratio**  **(95% CI)** | **p value** |  | **Hazard Ratio**  **(95% CI)** | **p value** | **C-statistics**  **(95% CI)** |
|  |  |  |  |  |  |  |
|  |  |  |  |  |  |  |
| Endo-RSS <50% | 1.38 (0.91-2.09) | 0.13 |  | 1.32 (0.90-2.09) | 0.61 | 0.73 (0.68-0.77) |
| Mid-RSS <50% | 1.69 (1.14-3.17) | **<0.001** |  | 1.71 (1.13-3.19) | **<0.001** | 0.74 (0.70-0.78) |
| Epi-RSS <50% | 2.01 (1.25-3.22) | **<0.001** |  | 1.97 (1.20-3.20) | **<0.001** | 0.77 (0.73-0.79) |
| Intramyocardial-RSS <50% | 2.12 (1.38-3.55) | **<0.001** |  | 2.03 (1.33-3.49) | **<0.001** | 0.78 (0.74-0.80) |
|  |  |  |  |  |  |  |
|  |  |  |  |  |  |  |

Adjusted model 2 included: diuretics, beta-blockers, antiarrhythmic agents, calcium channel blockers (CCB), angiotensin-converting enzyme (ACE) inhibitors, angiotensin receptor blocker (ARB), antiplatelet or anticoagulation agents, and one by one the variables of interest among: global circumferential strain, Endo-RSS, Mid-RSS, Epi-RSS, Intramyocardial-RSS or LVEF.

Abbreviations: *same abbreviations as Table 1* + RSS: regional strain score.

**SUPPLEMENTARY FILE 8:**

**Determination of the optimal cut-off to transform Endo-, Mid-, Epi- and Intramyocardial-regional strain score (RSS)** **into a binary variable with the best predictive value for each outcome.**

The survival tree method was used to identify the cut-off to transform Endo-, Mid-, Epi- and Intramyocardial-RSS into a binary variable with the best predictive value:

- The best Endo-RSS cut-off to predict incident HF was 53.3% and to predict hard CHD 54.1%.
- The best Mid-RSS cut-off to predict incident HF was 48.3% and to predict hard CHD 48.9%.
- The best Epi-RSS cut-off to predict incident HF was 49.2% and to predict hard CHD 51.7%.
- The best Intramyocardial-RSS cut-off to predict incident HF was 50.2% and to predict hard CHD 51.6%.

Therefore, the mean best cut-off for all RSS to predict incident HF was 50.3%, and to predict hard CHD 51.6%


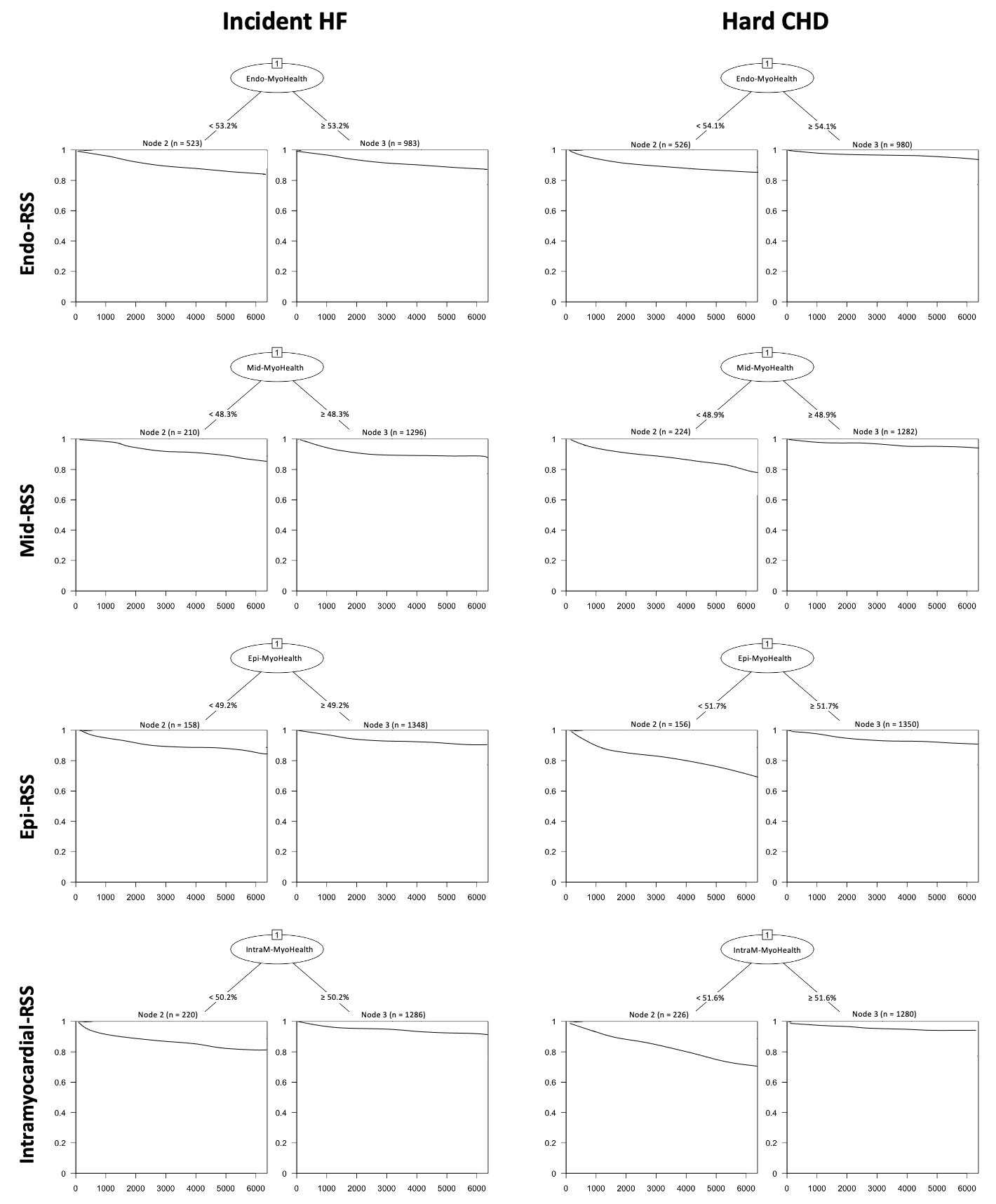


**SUPPLEMENTARY FILE 9:**

**Univariable and Multivariable Analysis for Hard CHD for model 2 (N=1,506).**

|  | **Univariable analysis** | |  | **Multivariable analysis** | | |
| --- | --- | --- | --- | --- | --- | --- |
|  | **Hazard Ratio**  **(95% CI)** | **p value** |  | **Hazard Ratio**  **(95% CI)** | **p value** | **C-statistics**  **(95% CI)** |
|  |  |  |  |  |  |  |
|  |  |  |  |  |  |  |
| Endo-RSS <50% | 1.33 (1.22-1.59) | **<0.001** |  | 1.45 (1.11-1.67) | **<0.001** | 0.73 (0.69-0.77) |
| Mid-RSS <50% | 1.85 (1.32-2.31) | **<0.001** |  | 1.83 (1.29-2.61) | **<0.001** | 0.74 (0.70-0.77) |
| Epi-RSS <50% | 2.32 (1.56-3.60) | **<0.001** |  | 2.06 (1.47-3.49) | **<0.001** | 0.74 (0.70-0.78) |
| Intramyocardial-RSS <50% | 2.40 (1.60-3.74) | **<0.001** |  | 2.24 (1.47-3.46) | **<0.001** | 0.75 (0.71-0.79) |
|  |  |  |  |  |  |  |
|  |  |  |  |  |  |  |

Adjusted model 2 included: diuretics, beta-blockers, antiarrhythmic agents, calcium channel blockers (CCB), angiotensin-converting enzyme (ACE) inhibitors, angiotensin receptor blocker (ARB), antiplatelet or anticoagulation agents, and one by one the variables of interest among: global circumferential strain, Endo-RSS, Mid-RSS, Epi-RSS, Intramyocardial-RSS or LVEF.

Abbreviations: *same abbreviations as Table 2.*
